# Assessment of Impact of the Surface Modification Techniques on Structural, Biophysical, and Electrically Conductive Properties of Different Fabrics

**DOI:** 10.3390/ma17051169

**Published:** 2024-03-02

**Authors:** Ewa Skrzetuska, Adam K. Puszkarz, Justyna Nosal

**Affiliations:** Textile Institute, Faculty of Material Technologies and Textile Design, Lodz University of Technology, 116 Zeromskiego Str., 90-924 Lodz, Poland; justynapytka@gmail.com

**Keywords:** biophysical comfort, thermal insulation, flocking, screen printing, thermal-transfer printing, layer by layer, fabric, clothing, micro-CT

## Abstract

This article presents studies on the evaluation of the impact of surface modification of cotton, viscose, and polyester fabrics using three techniques (flocking, layer by layer, and screen printing) with materials with electrically conductive properties on their structural, biophysical, and conductive properties. Each tested fabric is characterized by specific biophysical properties. which can be disturbed by various modification methods, therefore, the following tests were carried out in the article: optical microscopy, micro-computed tomography, guarded perspiration heating plate, air permeability, sorption and electrical conductivity tester. The use of screen printing increased the thermal resistance of the cotton woven fabric by 119%, the polyester woven fabric by 156%, and the viscose fabric by 261%. The smallest changes in thermal resistance compared to unmodified textiles were observed in layer by layer modified fabrics and are as follows: −15% (cotton woven fabric), +77% (PES woven fabric), and +80% (viscose woven fabric).

## 1. Introduction

Surface modification is one of the most intensively researched technological issues, which is related to almost all branches of industry, which also includes textronics. Textronics is a relatively new field of science that has emerged from areas such as textiles, electronics, computer science, and metrology. It is a dynamically developing field, and it owes its dynamic development thanks to the intensive development of fabric construction, textile technology as well as the constantly expanding range of electronic circuit applications. Textronic products integrate miniaturized electronics and electronic systems with fabric into a functional whole. The most common use of textronics and its typical applications are intelligent protective and utility products, but it can be found more and more often in everyday life, primarily in the field of medicine, sports and health care [1,2,3,4].

The systems built into the clothing structure are to perform their functions without disturbing the user’s comfort (physiological and physical balance). This is possible thanks to the use of properties characteristic of some raw materials from which intelligent products are made, i.e., piezoelectric, shape memory and electrostrictive properties. Their properties change under the influence of an external stimulus, which may be a chemical, mechanical or electrical stimulus [5,6,7,8,9,10].

Printing techniques are increasingly used in the creation of sensors. Prints on a textile substrate made using pastes or inks, for example based on nanotubes, bring many new, interesting applications. Printed sensors are much more convenient to use, more flexible, do not cause embarrassment, and are sensitive and give adequate measurement results [11,12].

Textronic elements of clothing can be used to monitor vital functions, such as respiratory rate, heart rate, pulse, without limiting the user’s comfort and body performance. In order to adequately monitor the physiological processes of the body, it is extremely important to deploy the sensors using clothing construction techniques. Textronic elements are used in the creation of wireless networks and systems for monitoring physiological signals during everyday activities, as well as supporting the detection of threats in our environment [8,13,14].

Scientists from the Lodz University of Technology have developed biodegradable non-woven fabrics made of melt-blown polymer, which react to the vapors of toxic substances. Non-woven fabrics were made of 98% polylactide and 2% MWCNT carbon nanotubes, their electrical resistance oscillates at the level of 10^6^ Ω. They are used as a filter material in respiratory protection against toxic particles. The solution developed at the Lodz University of Technology can be used to create sensors that monitor the concentration of toxic solvent vapors in the inhaled air [4].

The same research team also produced polyethylene oxide (PEO) nonwoven fabrics with 3% carbon nanotubes added using an electrospinning method. These nonwoven fabrics react to a chemical stimulus in the form of solvent vapors with a concentration of 100 ppm [4]. Fiber sensors are used as sensors to detect dangerous concentrations of chemical substances in protective clothing. Scientists under the direction of Professor Krucińska from the Lodz University of Technology have also developed a prototype nonwoven fabric that reacts to a thermal stimulus. They are based on a polymer composition of polycaprolactone, polypropylene and carbon nanotubes. The conducted research confirms the possibility of their use in the production of temperature-sensitive sensory materials [4].

The first prototypes of clothes for monitoring vital signs were created as part of the Wearable Health Care System project. Electrically conductive and piezoresistive fibers were used to produce sensors. The task of the system was to support patients during rehabilitation or people working in extreme environmental conditions by monitoring physiological activities in real time. Using the system, it was possible to record parameters such as breathing, body temperature, movement and electrocardiography [15]. A group of scientists from the Spanish university Universidad Carolos III de Madrid propose a similar solution. They developed a smart T-shirt that looks like a regular T-shirt but is fully equipped with sensors that monitor heart rate, temperature, and even allows you to perform an ECG (Electrocardiography) test remotely. The data is sent to the monitoring station, where it is analyzed by doctors [16].

Another example of a smart T-shirt is the Chronius project—designed specifically for patients with Chronic Kidney Disease (CKD) and Chronic Obstructive Pulmonary Disease (COPD) and adaptable to monitor other chronic conditions that require long-term care [17].

The growing interest in textronic solutions and the use of printing technology to create sensors became the reason to undertake this topic of work. The ability to conduct electrical impulses was an essential feature that the material had to have in the case of the work being carried out. The use of conductive pastes as printing compositions allowed to achieve the desired effect. Electrically conductive pastes contain electrically conductive compounds in the form of nanoparticles of gold, silver, copper metals or carbon compounds such as graphite or nanotubes [5,6,8]. Currently, a very wide range of conductive pastes are available on the market. An example is Carbon Conductive Assembly Paste MG CHEMICALS 847, designed to improve electrical contact between rough surfaces. It does not delaminate and does not spray under the influence of high temperatures and contains special corrosion inhibitors [18].

The next group of conductive materials are inks, produced for example by Amepox. One of such products is NANO INK AX JP-60n containing 20 to 40% of silver nanoparticles. Its main application is the printing of conductive circuits using the Ink-Jet method on foils [19]. Nanocyl is another example of a company producing numerous conductive materials based on carbon nanotubes. Their offer includes pure carbon nanotubes NC7000™, elastomeric concentrate, and water dispersions [20].

Scientists from the Lodz University of Technology proposed the introduction of chemical sensors directly onto textile products as electrically conductive paths using the screen-printing method. The tests assessed the sensitivity of textile substrates printed with an ink composition based on carbon nanotubes to a chemical agent in the form of liquids and vapors. The chemical stimulus causes changes in the electrical resistance of carbon nanotubes, and they themselves show chemo-sensory properties. The performance of the sensors on chemical stimuli was tested on selected liquids and vapors. Studies have shown that the best sensory properties were obtained for vapors of polar liquids, whose *R*_rel_ (Relative resistance) level was over 40%. The sensory sensitivity for non-polar liquids was much weaker and the *R*_rel_ level was about 25%. The printed textiles showed an immediate reaction to the chemical agent in the form of a liquid. In the case of vapors of these liquids, the reaction took place after a few seconds. Detection of dangerous chemical substances, such as the aforementioned organic liquids and their vapors, was possible thanks to the correct design of the sensors [7].

In this article, the authors decided to focus on assessing the impact of modification processes aimed at obtaining textronic products on their functional properties. It was decided to carry out comprehensive tests of structural properties (microtomography and microscopic examination), biophysical properties (air permeability, heat resistance, and water vapor resistance) and electrical conductivity. The research was carried out for three techniques of modification of textile substrates used in the textile industry, such as screen printing, flocking, and layer by layer. Three substrates differing in raw material composition were selected. The aim of this work was to assess whether each of the selected techniques will have a similar effect on the properties of substrates made of natural, synthetic and artificial fibers. The authors set themselves a goal, which was to answer the question whether each of the discussed techniques can be applied to any type of raw material used for textronic solutions. The most important aspect was to verify whether the techniques discussed would be suitable for creating sensors, i.e., whether they would have the ability to conduct electrical impulses, both before and after operational processes. This is a direction of research that will answer questions related to the durability of textronic products in everyday use.

## 2. Materials and Methods

### 2.1. Materials

**Figure 1:**
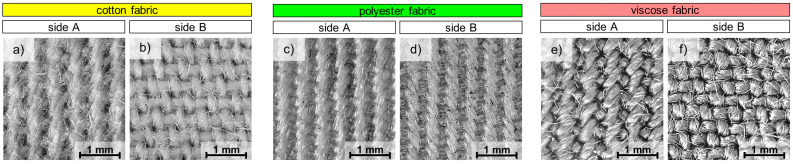
Optical microscopy images of both sides of tested fabrics: cotton (**a**,**b**) polyester (**c**,**d**) and viscose (**e**,**f**).

The subject of the tests were three woven fabrics made of cotton, polyester, and viscose. The physical parameters of all the fabrics are shown in Table 1, while in Figure 1 optical microscopy images of both sides of the fabrics are shown. All tested fabrics had the same weave (twill) and similar thickness, surface mass, and yarn porosity. The choice of cotton, polyester, and viscose as textile raw materials resulted from the need to verify the modification of textiles made of different types of fibers (natural, synthetic, and artificial). Test samples were prepared in sizes 32 cm × 32 cm, 3 identical samples from each tested variant.

### 2.2. Methods

#### 2.2.1. Modifications Method

##### Flocking

**Figure 2:**
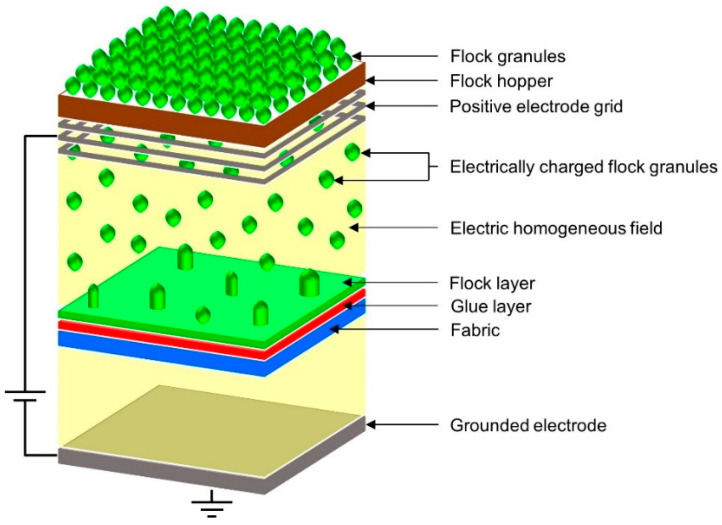
Scheme of applied flocking method.

In the flocking method, the electrostatic field accelerates and spatially orients electrically charged solids (short textile fibers or powder) deposited on an adhesive substrate (the diagram of the method is shown in Figure 2). The flocking is used to modify the surface of textiles, wood, paper, foil, plastic, metal, plaster, glass, cardboard, concrete, rubber, and others. The deposited flock layer is antistatic, abrasion-resistant, sound-absorbing, resistant to washing and cleaning, has thermal insulation and filtration properties. A full description of the method used was presented in the authors’ previous publication [23].

##### Layer by Layer

**Figure 3:**
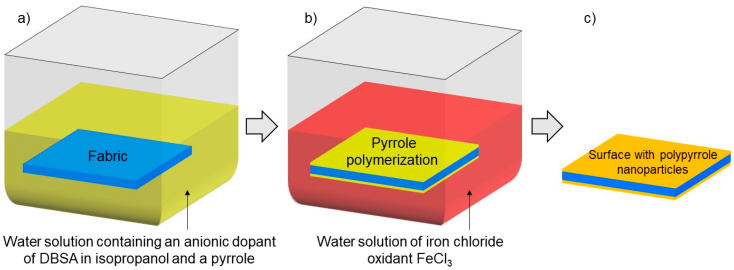
Scheme of applied layer by layer chemical method (**a**–**c**).

Layer by layer (Figure 3) is one of the most commonly used methods of surface modification in order to give them specific functional properties. Compared to other methods of surface modification, the layer by layer method has many key advantages, such as ease of use, low cost, independence from the size, and shape of the substrate [23]. Full characteristics of the layer by layer method used were presented in the authors’ previous publication [23].

##### Screen Printing

**Figure 4:**
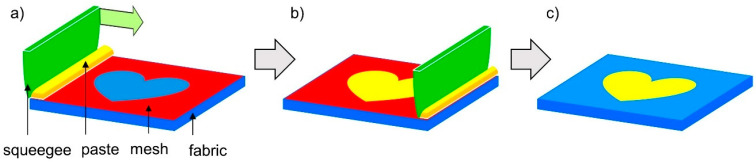
Scheme of screen printing (**a**–**c**).

Screen printing is a very durable, extremely durable method of printing on the surface of various materials. This method consists of forcing the ink into the material through a printing form, thus creating a pattern on the substrate (Figure 4). Screen printing ensures high color durability and resistance to washing and abrasion. This technology is very popular as a method of decorating light clothing, such as T-shirts or pajamas, functional clothing, but it is also used to modify surfaces other than clothing textiles [23,24,25,26,27]. Full characteristics of the method used were presented in the authors’ previous publication [23].

#### 2.2.2. Evaluation of Modified Fabrics Properties

##### Structural Properties

*Optical microscopy (OM) and X-ray micro-computed tomography (micro-CT)*

**Figure 5:**
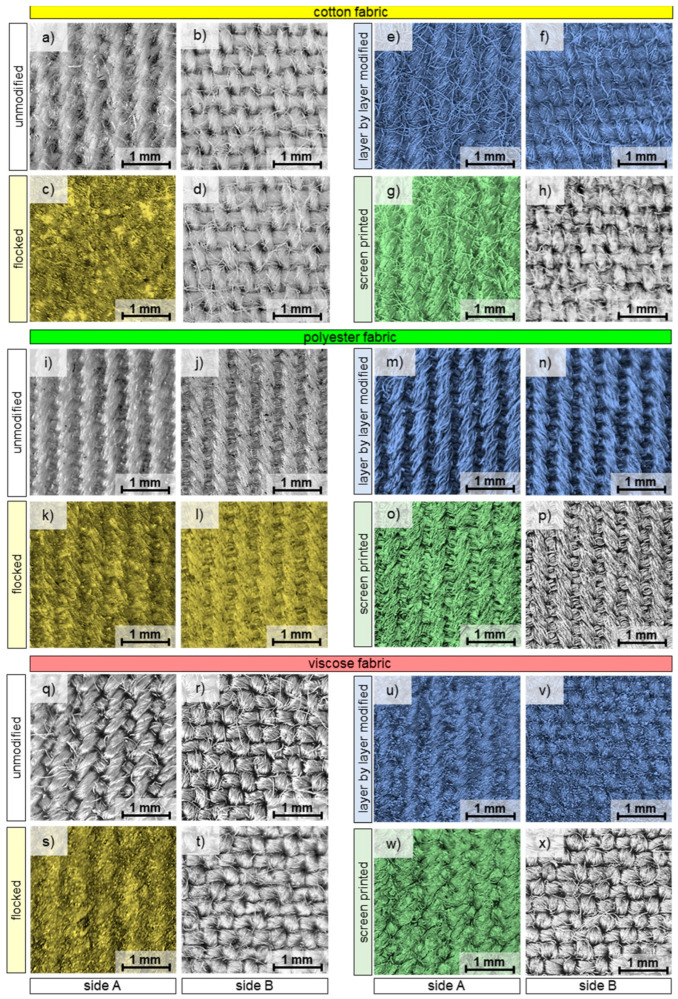
Optical microscopy images of both sides of unmodified and modified tested textiles: cotton fabric (**a**–**h**), polyester fabric (**i**–**p**), viscose fabric (**q**–**x**).

Optical microscopy was used to determine selected geometrical parameters of the tested woven fabrics and to characterize their surface before and after modification using three different techniques. The images shown in Figure 1 and Figure 5 were taken using an optical microscope Delta Optical Smart 5MP PRO (Delta Optical, Warsaw, Poland) and software Delta Optical Smart Analysis Pro 1.0.0.

High-resolution X-ray tomography (micro-CT, SkyScan 1272; Bruker, Kontich, Belgium) was used to obtain the characteristics of the structure of the tested textiles and to examine the effect of the three types of applied surface modifications on the structure of the woven fabrics. The following scanning conditions were used: X-ray source voltage 50 kV, X-ray source current 200 µA, pixel size 5 µm. A 180° rotation was performed with a rotation step of 0.1° and no filter was selected.3D reconstruction of the tested textiles were obtained using NRecon 1.7.4.2 and CTvox 3.3.0 r1403 software made by Bruker. Geometrical parameters of woven fabrics were calculated using CTAn 1.17.7.2+ and Data Viewer 1.5.6.2 software made by Bruker.

##### Biophysical Properties

*Thermal resistance, water vapor resistance, air permeability and sorption properties*

In order to investigate the effect of three types of modifications on thermal resistance (*R*_ct_) and water vapor resistance (Ret) of tested woven fabrics, both biophysical parameters were measured using a Sweating guarded Hotplate 8.2 (made by Measurement Technology Northwest in Seattle, WA, USA) according to PN-EN ISO 11092:2014-11, ISO 7243 [28,29,30]. The effect of the applied modifications on the air permeability of the tested woven fabrics was measured using the method described in PN-EN ISO 9237:1998 [31] using an air permeability tester (FX 3300, Textest Instruments, Schwerzenbach, Switzerland). Tests of material samples were carried out in normal climate conditions in accordance with the PN EN ISO 139:2006 standard [32], for which the air temperature is 20 ± 2 °C and the relative air humidity is 65 ± 4%. The samples were previously conditioned for 24 h under the above conditions. The tests were carried out on samples measuring 32 cm × 32 cm. For thermal resistance, 3 tests were performed for each variant, for air permeability and sorption, 10 measurements were performed in different places [32]. A more detailed description of the methods used was presented in the authors’ earlier work [23]. To determine the sorption properties, 10 disk-shaped samples with an area of 15.73 cm^−2^ (diameter of the die) were cut out of the tested materials, and then weighed. Sorption properties were determined on the SORP3 apparatus. Each of the samples were successively placed on the holder, which was also the weight of the sample. Then, the samples were introduced into the measuring cup, where the cut discs were in contact with the Schott plate (sinter). At this point, the water layer was absorbed by the fibers and/or pores of the sample. Under the sinter, the water pressure (registered by the sensor) decreased, which was equalized thanks to the pump. The appropriate program calculated the amount of absorbed liquid, additionally the speed of the pump operation allowed to calculate the speed of sorption. In this study, the maximum sorption rate in µL·cm^−2^ (*V*_max_), the maximum sorption rate in µL·cm^−2^ (*S*_max_), the initial time in s, (*t*_0_), the total sorption time in s, (*t*_max_) were determined. The sorption capacity was determined from the Formula (1):

(1) $$d=\frac{S_{max}\times 10}{M_{p}}$$

where: *d*—sorption capacity; µL·cm^−2^*; M_p_*—surface mass of the sample, g·m^−2^.

##### Electrically Conductive Properties

*Surface resistivity*

To investigate how the three applied modifications affect the electrical properties of the woven fabric surface, measurements of the surface resistance were made in accordance with PN-EN ISO 1149-1 Protective clothing—Electrostatic properties—Part 1: Surface resistivity [33]. Tests of material samples were carried out in normal climate conditions in accordance with the PN EN ISO 139:2006 standard [32], for which the air temperature is 20 °C ± 2 °C and the relative air humidity is 65% ± 4%. The samples were previously conditioned for 24 h under the above conditions. The tests were carried out on samples measuring 32 cm × 32 cm. For thermal resistance, 3 tests were performed for each variant, and 10 measurements were made in different places [32].

## 3. Results

### 3.1. Structural Properties

In Figure 5, optical microscopy images of both sides of the unmodified and modified woven fabrics are shown. In the case of modified woven fabrics, selected photos have been colored (the color is not related to the substance used to modification) in order to show that the applied modifications caused changes on both or one side of the modified fabric. As can be seen from the images, the only type of modification that affected both sides of the woven fabric were the layer by layer technique, although the effects of flocking were also observed in the case of polyester woven fabric.

In the case of flocked woven fabrics, the images of the modified side (Figure 5c,k,s) show the granulate layer deposited on the glue layer. The glue and granulate formed a layer thick enough to prevent identification of the woven fabric weave. The indistinct outline of the weave is visible in the case of polyester and viscose woven fabrics, while completely invisible in the case of cotton woven fabrics. In the case of the other two woven fabrics, probably due to the greater hydrophilicity of cotton and viscose than polyester, the glue did not penetrate to the unmodified side (Figure 5d,t). The presented optical microscopy images also showed that the layer by layer modification of all three woven fabrics is double-sided and both sides of the textiles have been modified at a comparable level. On the other hand, the effect of screen printing was observed only on one side of the textiles, and it is at a similar level for all three tested woven fabrics.

**Figure 6:**
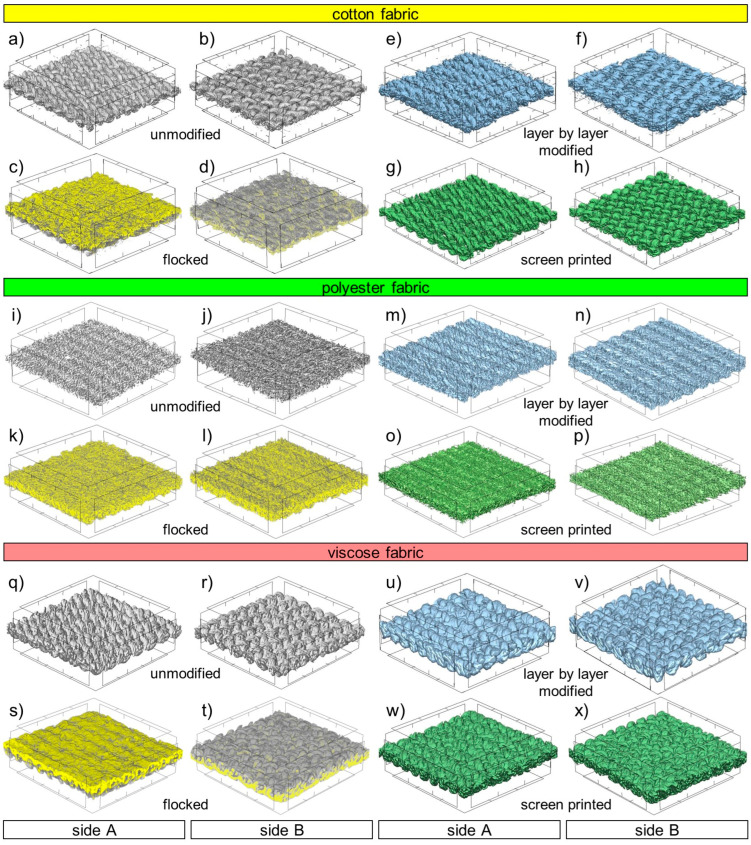
3-D micro-CT reconstruction of both sides of unmodified and modified tested textiles: cotton fabric (**a**–**h**), polyester fabric (**i**–**p**), viscose fabric (**q**–**x**)—for all textiles surface was reduced to dimensions 3 mm × 3 mm.

**Figure 7:**
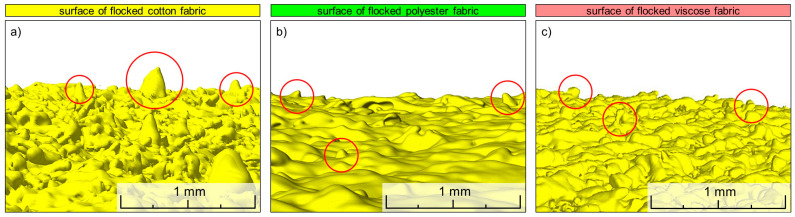
3D micro-CT reconstruction of flocked surface of tested textiles: (**a**) cotton fabric, (**b**) polyester fabric, (**c**) viscose fabric. Selected objects made of stuck granules formed flocked fabric surface in the form of cones oriented vertically with the tip up were indicated inside the red circles.

In addition, to compare the effects of the applied modification methods on the spatial structure of the tested woven fabrics, 3D reconstructions of unmodified and modified textiles using computer microtomography were performed (Figure 6). The reconstructions show both sides of fabrics with the surface reduced to a square with sides 3 mm × 3 mm. In the case of all three tested woven fabrics, the flocking effect is the most pronounced among all the modification methods used. Since the layer of glue and granules clearly differed from cotton, polyester, and viscose in terms of X-ray absorption, it was possible to mark the modifying layer with a different color (yellow) than textiles (gray). Based on the obtained reconstructions, it can be observed that in the case of cotton and viscose, only one side was modified as a result of flocking (Figure 6c,d,s,t). In the case of the more water-repellent polyester woven fabric, the glue has migrated to the other side of the woven fabric (Figure 6k,l). The flocked side of the fabrics, as a result of being covered with a continuous layer of glue, was almost completely non-porous, and as a result of the applied electrodeposition, objects built of glued granules in the form of vertically oriented cones, with the top pointing up, formed on its surface. The cones were shown on a larger scale in Figure 7.

Depending on the type of substrate (woven fabrics with different raw material composition), the number of cones per unit of surface varies. The largest surface concentration of cones was observed in the case of cotton fabric, and the smallest in the case of polyester fabric. Moreover, the height and shape of the cones is also different for each of the three tested fabrics. The highest and most elongated ones were observed on the surface of cotton fabric. The cones on the surface of the viscose woven fabric are lower and have a less elongated shape. On the other hand, on the surface of the polyester woven fabric, the cones are the lowest and most flattened, slightly protruding above the surface of the flock layer. From Figure 7, it can also be seen that the flock surface is the least developed (smooth) for the polyester woven fabric and the most developed (rough) for the cotton woven fabric. This is probably related to the degree of hydrophobicity of the flocked woven fabrics. In the case of the most hydrophobic polyester woven fabric, the glue layer did not stop on the modified side and partially penetrated to the other side, leveling the surface irregularities resulting from the weave of the woven fabric. In the case of the least hydrophobic cotton woven fabric, the glue did not penetrate to the other side of the woven fabric and increased the level differences between different areas of the woven fabric surface. For all three fabrics modified with layer by layer, the modification effect was observed on both sides of the textile, although it was clearly stronger on the side B (Figure 6f,n,v). This is probably the result of the side A of the modified fabric adhering to the smooth substrate, which hindered the migration of nanoparticles to the other side of the woven fabric. For all three tested woven fabrics, the screen-printing effect is one-sided. The modified B side has a much thicker yarn due to the absorption of paste (Figure 6h,p,x). In addition, all three screen printed woven fabrics are characterized by a less fluffy structure caused by the pressure of the squeegee. In general, based on three-dimensional reconstructions, it can be observed that the effect of all applied modifications caused the largest changes in spatial geometry of cotton woven fabric (the most hydrophilic), and the smallest in the case of polyester woven fabric (the most hydrophobic).

**Figure 8:**
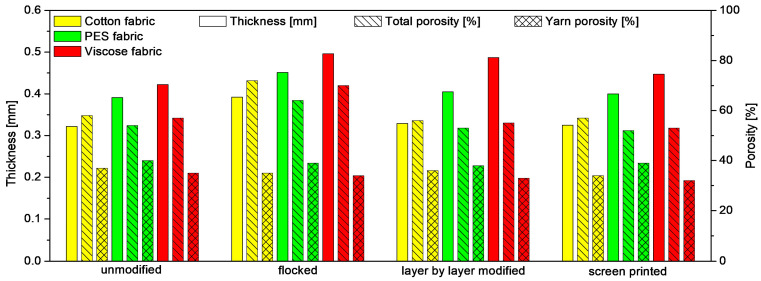
Structural parameters of unmodified and modified fabrics.

Figure 8 show the effect of the applied modifications on the structural parameters (thickness, porosity, and yarn porosity) of the three tested fabrics. Based on the presented results, it can be observed that each type of modification increases the thickness of the woven fabrics, regardless of the raw material from which they were made. The exception is screen printed woven fabric made of cotton, the thickness of which has decreased compared to the unmodified cotton woven fabric. This is probably due to the fact that the cotton woven fabric is less flexible than the other two fabrics (PES and viscose) and has deformed more due to the squeegee pressure during modification. The greatest increase in thickness in relation to the unmodified fabric was observed in the case of fabrics modified with the use of the flocking technique (cotton fabric: +22%, PES fabric: +15%, viscose fabric: +18%). For all fabrics, the flocking technique caused the greatest increase in total porosity, with the largest increase in relation to the unmodified textile (+24%) was observed in the case of cotton fabric, which is the least hydrophobic of all the tested fabrics. All three applied modifications cause a decrease in yarn of woven fabrics compared to unmodified textiles and ranged from −2.5% (flocked woven fabric and screen-printed PES woven fabric) to −8.6% (screen printed viscose woven fabric).

### 3.2. Biophysical Properties

**Figure 9:**
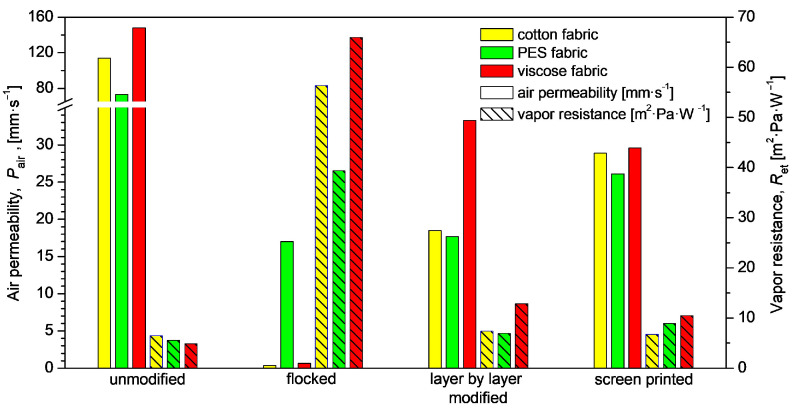
Air permeability and vapor resistance of unmodified and modified fabrics.

**Figure 10:**
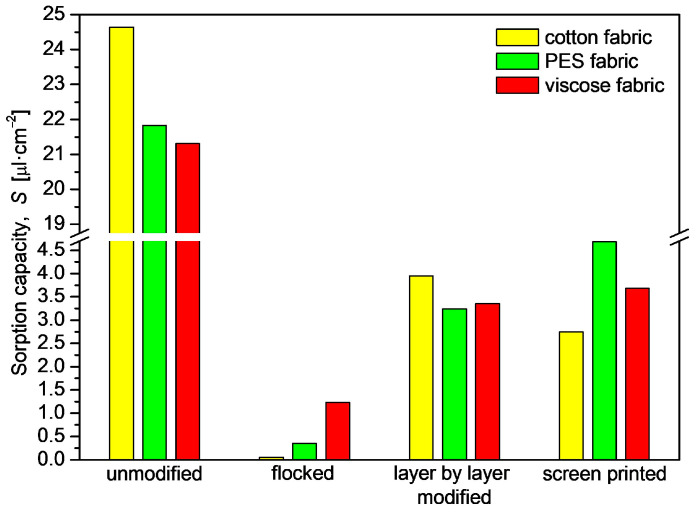
Sorption capacity of unmodified and modified woven fabrics.

**Figure 11:**
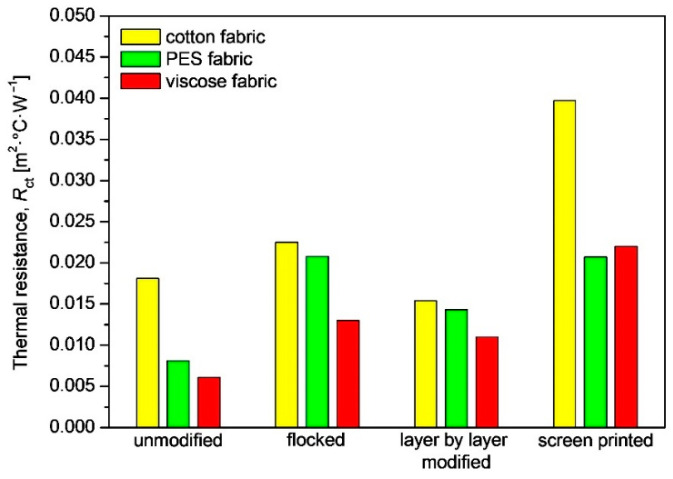
Thermal resistance of unmodified and modified woven fabrics.

The applied modifications also had an impact on the biophysical properties of the tested woven fabrics. Figure 9, Figure 10 and Figure 11, respectively, show the air permeability and water vapor resistance, sorption capacity, and thermal resistance of unmodified and modified woven fabrics. The greatest changes in air permeability, compared to unmodified textiles, were observed in the case of flocked fabrics, where the applied layer of glue and granules most effectively reduce the surface clearance of the modified fabrics.

In the case of modified fabrics: cotton and viscose, greater changes were observed than in the case of polyester fabric, where the layer of glue and granules partially penetrated to the other side of the material, so that an airtight layer was not formed on the modified side. In the case of cotton fabric, the flocking method reduced the air permeability by 99.7% compared to the fabric of unmodified textile, while in the case of polyester woven fabric by 76.8%. The smallest changes in air permeability compared to unmodified textiles were observed for screen printed fabrics, where air permeability was reduced between 64% and 80% depending on fabric raw material. As with air permeability the greatest changes in vapor resistance compared to unmodified textiles were observed in the case of flocked woven fabrics, where a layer of glue and granules created the most effective barrier to water vapor. The use of flocking resulted in the following increase in water vapor resistance compared to unmodified textiles: +869% (cotton woven fabric, +712% (PES woven fabric) and +1349% (viscose woven fabric). The smallest increase in vapor resistance compared to unmodified textiles were noticed in the case of screen-printed woven fabrics: +4% (cotton woven fabric), +60% (PES woven fabric) and +113% (viscose woven fabric). This may be due to the fact that this method has the least effect on the swelling of the fibers and, as a result, does not seal the material as much as the other two methods of modification.

Cotton fabric (24.645 µL·cm^−2^) has the highest sorption capacity among non-modified materials, while viscose fabric has the lowest (21.313 µL·cm^−2^). The lowest values were obtained for fabrics after the flocking process.

The greatest changes in thermal resistance compared to unmodified textiles were observed in the case of screen-printed woven fabrics. This is dictated by the fact that the spaces between the fibers of the fabric have been clogged and flattened, resulting in an additional layer of insulation resulting from the applied printing paste containing carbon nanotubes, which are characterized by high thermal insulation. The use of screen printing increased the thermal resistance of the cotton woven fabric by 119%, the polyester woven fabric by 156%, and the viscose fabric by 261%. The smallest changes in thermal resistance, compared to unmodified textiles, were observed in the case of layer by layer modified fabrics and are as follows: −15% (cotton woven fabric), +77% (PES woven fabric) and +80% (viscose woven fabric).

### 3.3. Electrically Conductive Properties

**Figure 12:**
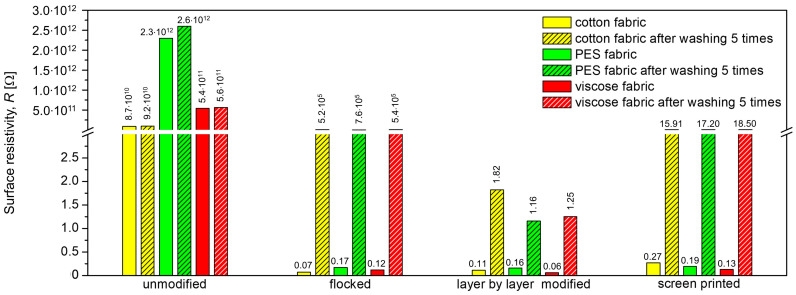
Surface resistivity of unmodified and modified woven fabrics.

Figure 12 shows the influence of the applied modifications on the surface resistivity of the three tested woven fabrics. Based on the presented results, it can be seen that each of the three applied techniques makes the modified fabric surface electrically conductive.

As a result of the applied modifications, the surface resistance decreased by 11–12 orders of magnitude, depending on the technique and woven fabric raw material. In the case of cotton woven fabric, the most effective technique in imparting electrically conductive properties was flocking (0.07 Ω), in the case of polyester woven fabric and viscose woven fabric—layer by layer (0.16 Ω and 0.06 Ω, respectively). The highest resistance values were observed for samples with a paste layer based on nanotubes. In the case of cotton, this value was 0.27 Ω. Lower resistance values obtained for samples modified by the in situ method are associated with a different degree of polypyrrole conversion on the textile substrate, while the values obtained for flocked samples depend to a large extent on poor adhesion of activated carbon particles. The conservation process of 5 washing cycles had a negative impact on the conductive properties of the modified textile substrates. The smallest changes are observed for modifications using the layer by layer method, while the largest changes occur for the flocking method. On this basis, it can be claimed that the conductive particles in the layer by layer technique are permanently bonded to the textile substrate. In the case of the screen printing method, a slight deterioration of the conductive properties was observed, which may be related to the damage of microcracks in the conductive paths created during conservation. In the case of flocking, the conductive fibers became detached during the conservation process, as a result of which the conductivity deteriorated significantly. Tests on the surface resistivity of material samples were carried out in normal climate conditions in accordance with the PN EN ISO 139:2006 standard, for which the air temperature is 20 °C ± 2 °C and the relative air humidity is 65% ± 4%. The samples were previously conditioned for 24 h under the above conditions.

## 4. Discussion

Analyzing the data, the largest increase in surface weight was observed for flocked samples. In this case, for each of the fabrics, changes occurred at the level of over 50% compared to the starting samples. The largest increase was noted for the viscose fabric sample, where the weight increased by 124.8 g·m^−2^. These results are closely related to the application of a layer of glue and flock.

Fabrics modified by the in situ method are distinguished by the greatest thickness. Similar to the surface weight, the increase in thickness of these samples was more than 50% compared to the initial fabrics. Among them, the cotton sample with 0.65 mm has the greatest thickness. The in situ method was based on immersing the samples in aqueous solutions (including DBSA acid), which could have affected the swelling of the fibers, leading to an increase in their thickness. A decrease in thickness was noted in the case of cotton fabric modified with the film printing method, this result is associated with the strong pressure of the squeegee.

The values obtained for the thermal resistance show that the greatest increase was observed for the cotton sample coated with the printing composition based on nanotubes. The average values obtained for the initial sample were 0.0181 m^2^·K·W^−1^, while after modification this value increased to 0.0397 m^2^·K·W^−1^. It can be seen that applying layers to the substrates of the analyzed fabrics led to an increase in their thermal resistance. The ink composition and the adhesive layer clogged the fissures and pores of the fabrics, while the in situ method led to swelling of the fibers, which is associated with greater heat retention.

The greatest increase in water vapor resistance was obtained by samples subjected to flocking, in particular viscose fabric, where the water vapor resistance for the starting sample was 4.8910 m^2^·Pa·W^−1^, while after applying the flock layer the value increased to 65.9579 m^2^·K·W^−1^. As a result of applying the glue, the pores were clogged, the fabric did not absorb water and did not let water vapor through.

Surface modification processes had a negative impact on air permeability. The greatest decrease was noted for the samples subjected to flocking, where the values for cotton and viscose decreased to a level close to zero. For pure cotton, this value decreased from 114 mm·s^−1^ to 0.35 mm·s^−1^. This method has a significant impact on the structure of fabrics. The adhesive particles clogged the voids and pores, making the fabrics virtually impermeable. A similar situation occurs in the case of film printing. Lower values were also recorded for the in situ method. As a result of fiber swelling, the permeability of the viscose fabric decreased by 114.4 mm·s^−1^ in relation to the starting sample. In addition, as the permeability decreases, the thermal resistance of the materials increases.

The processes of deposition of electrically conductive layers significantly decreased the sorption capacity of the tested fabrics. The lowest values were found in the samples subjected to flocking, where the sorption capacity of the starting cotton sample was 24.645 µL·cm^−2^, after applying the flock layer the value decreased to 0.054 µL·cm^−2^. In addition, for the viscose fabric modified with the film printing method, the sorption capacity decreased by 17.632 µL·cm^−2^ in relation to the starting sample. In these two cases, the application of additional layers caused the voids and pores to be plugged, so that the liquid had nowhere to penetrate. The decrease in the sorption capacity of the samples modified by the in situ method was due to the swelling of the fibers.

The highest resistance values were observed for samples with a paste layer based on nanotubes. In the case of cotton, this value was 0.27 Ω. Lower resistance values obtained for samples modified by the in situ method are associated with a different degree of polypyrrole conversion on a textile substrate, while the values obtained for flocked samples largely depend on poor adhesion of activated carbon particles.

## 5. Conclusions

The article presents research on the impact of three types of surface modification (flocking, layer by layer, screen printing) on three fabrics with similar spatial geometry and different raw material composition (cotton, polyester, viscose) on their biophysical structural and electroconductive properties. Based on the results obtained, the following conclusions can be drawn:

- Optical microscopy and X-ray microtomography showed that one side of the fabrics was modified as a result of flocking, while the use of layer by layer and screen printing techniques resulted in modification on both sides of the tested textiles.
- The results of X-ray microtomography showed that of all three modification techniques used, flocking resulted in the greatest increase in the thickness of all three tested fabrics. Moreover, as a result of flocking, the most even and smooth electroconductive surface was obtained on the most hydrophobic surface of the polyester fabric.
- The results of biophysical properties tests showed that of all the techniques used, flocking resulted in the greatest decrease in air permeability and sorption. On the other hand, the greatest increase in water vapor resistance compared to unmodified textiles. Additionally, the greatest increase in thermal resistance was observed for screen-printed textiles compared to unmodified fabrics.
- The results of testing the conductive properties of the fabrics showed that the use of the layer by layer technique provides the lowest surface resistance compared to unmodified textiles.

Based on the obtained test results, it can be concluded that when modifying textile substrates, the area of modification and their size on clothing products should be carefully selected so as not to negatively affect the feelings of potential recipients.

## Figures and Tables

**Table 1:** Physical parameters of tested fabrics.

Nr	Raw Material	Thickness ^(a)^ [mm]	Surface Mass ^(b)^ [g·m^−2^]	Total Porosity ^(c)^ [%]	Yarn Porosity ^(c)^ [%]
1	cotton	0.36	143.74	58	37
2	polyester	0.39	157.92	54	40
3	viscose	0.42	169.75	57	35

^(a)^ determined according to PN-EN ISO 5084:1999 [21]; ^(b)^ determined according to PN EN 12127:2000 [22]; ^(c)^ determined according to X-ray micro-CT.

## Data Availability

The data presented in this study are available on request from the corresponding author. The data are not publicly available due to privacy.
